# Magnetic resonance imaging at ultra-high magnetic field strength: An *in vivo* assessment of number, size and distribution of pelvic lymph nodes

**DOI:** 10.1371/journal.pone.0236884

**Published:** 2020-07-31

**Authors:** Ansje S. Fortuin, Bart W. J. Philips, Marloes M. G. van der Leest, Mark E. Ladd, Stephan Orzada, Marnix C. Maas, Tom W. J. Scheenen

**Affiliations:** 1 Department of Radiology and Nuclear Medicine, Radboud University Medical Center, Nijmegen, The Netherlands; 2 Department of Radiology, Ziekenhuis Gelderse Vallei, Ede, The Netherlands; 3 Medical Physics in Radiology, German Cancer Research Center (DKFZ), Heidelberg, Germany; 4 Faculty of Physics and Astronomy and Faculty of Medicine, University of Heidelberg, Heidelberg, Germany; 5 Erwin L. Hahn Institute for Magnetic Resonance Imaging, Essen, Germany; Henry Ford Health System, UNITED STATES

## Abstract

**Objective:**

The definition of an *in vivo* nodal anatomical baseline is crucial for validation of representative lymph node dissections and accompanying pathology reports of pelvic cancers, as well as for assessing a potential therapeutic effect of extended lymph node dissections. Therefore the number, size and distribution of lymph nodes in the pelvis were assessed with high-resolution, large field-of-view, 7 Tesla (T) magnetic resonance imaging (MRI) with frequency-selective excitation.

**Materials and methods:**

We used 7 T MRI for homogeneous pelvic imaging in 11 young healthy volunteers. Frequency-selective imaging of water and lipids was performed to detect nodal structures in the pelvis. Number and size of detected nodes was measured and size distribution per region was assessed. An average volunteer-normalized nodal size distribution was determined.

**Results:**

In total, 564 lymph nodes were detected in six pelvic regions. Mean number was 51.3 with a wide range of 19–91 lymph nodes per volunteer. Mean diameter was 2.3 mm with a range of 1 to 7 mm. 69% Was 2 mm or smaller. The overall size distribution was very similar to the average volunteer-normalized nodal size distribution.

**Conclusions:**

The amount of in vivo visible lymph nodes varies largely between subjects, whereas the normalized size distribution of nodes does not. The presence of many small lymph nodes (≤2mm) renders representative or complete removal of pelvic lymph nodes to be very difficult. 7T MRI may shift the in vivo detection limits of lymph node metastases in the future.

## Introduction

### Background

Determining the regional lymph node involvement in pelvic cancers is important for correct staging [1]. The presence of regional nodal metastases strongly influences prognosis and knowledge about their presence often influences therapy choice.

Diagnostic surgery in the form of an extended pelvic lymph node dissection has been historically regarded as the reference standard for imaging studies. Although this procedure is currently considered to represent the most accurate staging procedure, it has a considerable risk of adverse effects, while metastatic lymph nodes are not always all removed and the therapeutic effect is under debate [2–4].

### Current imaging

An in vivo evaluation of malignant lymph node involvement in the pelvic region is difficult. Conventional imaging techniques as Computer Tomography (CT) and Magnetic Resonance Imaging (MRI), based on size and shape show limited sensitivity and specificity in assessing the presence of metastases in lymph nodes [5–7]. Sensitivity can be increased by using small size criteria but at the cost of specificity, which can result in undesirable overtreatment [6,8].

Attempts have been made to define different upper size limits of normal lymph nodes for the different abdominal and pelvic territories [9]. The most recent study in this series demonstrated a 1.5 T MRI-determined threshold for suspicious mesorectal nodes of 4 mm and 6 or 7 mm for all other pelvic territories [10]. The mean sizes of normal lymph nodes in this study varied between 2.6 and 3.9 mm.

In rectal cancer there is a role for conventional MRI assessing morphology of lymph nodes with the purpose of differentiation between benign and metastatic lymph nodes [11]. For other pelvic cancers this is however not determined.

The role of 18F-FDG Positron Emission Tomography (PET) combined with CT or MRI is limited in pelvic carcinomas [12–15]. A functional imaging technique, ultrasmall superparamagnetic iron oxide (USPIO) enhanced MRI, showed a pooled sensitivity of 89% and a pooled specificity of 86% for the detection of lymph node metastases of pelvic tumours in a meta-analysis of Wu et al [16]. As this is a MRI-based technique, a spatial resolution superior to most other techniques can be obtained. However, the contrast agent used in this technique has not been available for a couple of years and only recently it has been re-introduced in the Netherlands [17].

Specifically for prostate cancer, another technique for the detection of (lymph node) metastases is ^68^Ga PET/CT or PET/MRI. Pooled sensitivity for lymph node detection of 61% and 74% and pooled specificity of 97% and 96% were reported for primary staging in two meta-analyses so far [18,19]. Initial results are promising, but the technique seems to have difficulties in detecting small lymph node metastases, while generally lymph node metastases in the pelvis tend to be limited in size [20,21].

### Surgery—pathology

In a surgery—histology study Schiavina et al [22] performed extended pelvic lymph node dissection (ePLND) and harvested 1064 lymph nodes in 54 patients with prostate cancer. The mean diameter of metastatic lymph nodes detected with standard pathological evaluation was 3 mm. Increasing the number of evaluated sections per lymph node (serial sectioning) and adding immunohistochemistry staining of the sections revealed more metastatic lymph nodes with smaller mean sizes of respectively 0,9 and 0.5 mm [22].

Improved histopathology shows lymph node metastases under the size thresholds defined by conventional imaging as mentioned above. This leads to a paradigm shift: it becomes less interesting what normal upper limits of normal lymph nodes are and more interesting how small lymph nodes (metastases) can be detected in vivo.

### Ultra-high magnetic field imaging

At an ultra-high magnetic field strength (7T), the increased sensitivity of MRI can enable three-dimensional MR examinations at a higher spatial resolution, therefore possibly visualizing smaller lymph nodes. Ex-vivo research has been showing promising results in mesorectal lymph node assessment at 7T. Many lymph nodes or lymph node like structures were found on ex vivo 7T MRI, with a small mean size [23].

To this end, a new approach has recently been introduced to use relatively straightforward gradient echo imaging designs with specifically chosen excitation pulse durations to discriminate between water and lipid signals, aiming to recognise lipid-embedded lymph nodes [24]. Typical challenges of 7T-related image inhomogeneities were overcome by the use of the time interleaved acquisition of modes technique (TIAMO) [25].

### Aim

In the current study we use ultra-high magnetic field MRI to determine the number, size and distribution of healthy lymph nodes in the pelvis to set a nodal anatomical baseline, in order to assess the detection limits of lymph node metastases in vivo for (future) functional imaging techniques.

## Materials and methods

### Subjects

Eleven healthy colleagues from our research department were included as volunteers. Mean age was 31 years (range 25–39), 10 male and 1 female. With a pre-study estimated average amount of MR visible lymph nodes of 25 in the pelvis of one subject, eleven volunteers would provide 275 visible lymph nodes. Divided over 6 areas in the pelvis we would have at least 40 lymph nodes in each region for characterizing amount and size of nodes per region. Exclusion criteria were primary pelvic disease, the existence of metal anywhere in the body and contraindications for Butylscopolamine.

The local institutional review board approved the study. All volunteers provided written informed consent prior to the MRI exam.

### MRI

After administration of 20 mg Butylscopolamine i.m. all volunteers were examined on a 7T whole body MR system (Magnetom 7T, Siemens Healthcare, Erlangen, Germany) with an 8 channel transceiver body-array coil with meander-type microstrip elements [24,25]. After B0 and B1+ shimming, water and lipid selective imaging was performed using the TIAMO technique to obtain homogeneous body imaging at ultra-high field [25,26]. The area of interest was the whole pelvis, scanning from the aorta bifurcation up to and including the pelvis floor.

Water-selective imaging was performed using a 3D multi Gradient Echo (GRE) sequence (repetition time (TR) 14 ms, voxel size 0.66x0.66x0.66 mm^3^, field-of-view (FOV) 210x210x169 mm^3^, matrix size 320x320x256, acquisition time (TA) 8 min 23 s, and multiple T2* weighted contrasts with echo times (TE) of 2.10, 4.19, 6.21, 8.30 and 10.32 ms), which was reconstructed at a TE of 6.21 ms [24]. Lipid selective imaging was performed with a similar, but single-echo 3D GRE sequence with identical FOV, matrix and voxel size (TE 2.09 ms, TR 5.2 ms, TA 2 min 51 s).

### Image analysis

The water and lipid selective image series were evaluated by 2 readers separately (ASF 8 years experience, ML 6 years experience or/and BP 2 years experience), after which consensus opinion was obtained by both readers together. The water-selective computed TE image set was used to detect and measure lymph nodes. The lipid-selective image set supported the distinction of lymph nodes from surrounding structures, for example blood vessels or ganglia.

Small confined areas of high signal intensity on the T2*-weighted water selective series, embedded in lipid tissue, were assessed and deemed lymph nodes after distinguishing them from ganglia or vessels by excluding structures on classical locations for ganglia [27] and excluding structures that were continuous or tubular on sequential images. To improve the assessment and help differentiate between small blood vessels and nodes, transversal, sagittal and coronal images could be viewed simultaneously and the readers were able to make thin maximum intensity projections (MIP).

After identification of all visible lymph nodes, the nodal sizes were measured, and their location marked by one reader (ASF). Size was defined as the short axis diameter in millimeters. The analysed anatomic locations were: 1) the common iliac artery region (CIA region); 2) the external iliac artery region (EIA region); 3) the internal iliac artery region (IIA region); 4) the presacral region; 5) the obturator region; 6) the pararectal region. Next to summarizing total numbers of detected lymph nodes and sizes, the relative nodal size distribution was calculated for each volunteer before averaging in an overall relative nodal size distribution.

## Results

**Fig 1:**
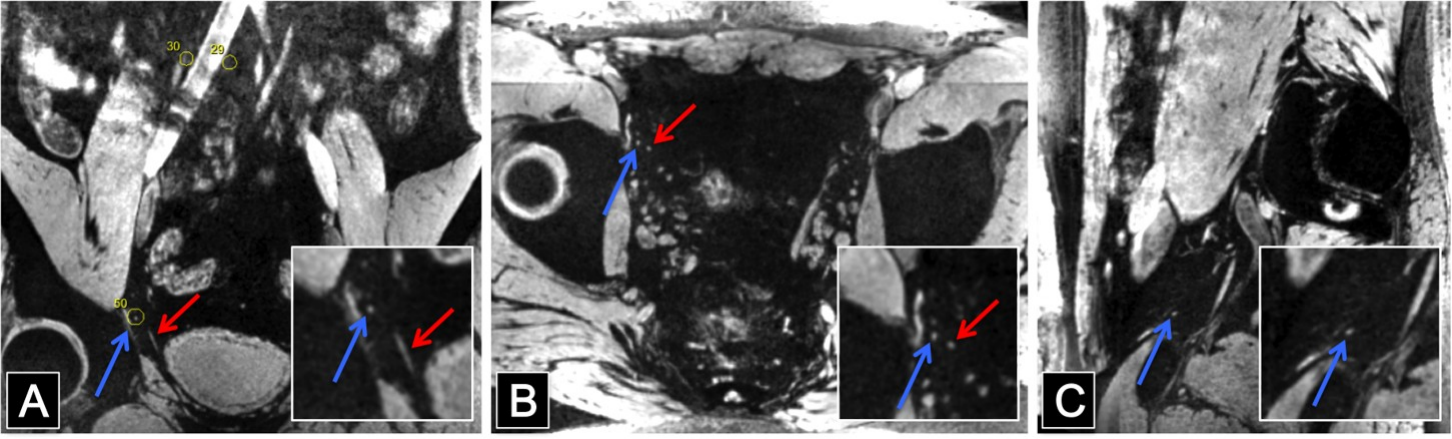
Water-selective images of the pelvis of a healthy volunteer. (A) Coronal image with magnified detail in box. Yellow circle around the detected lymph node. (B) Transversal image with detail box. (C) Sagittal image with detail box. In all images the blue arrow points at a small lymph node, the red arrow at a blood vessel.

In all volunteers the 7T MRI examinations were successfully concluded. All 3D multi GRE datasets were reconstructed into single computed TE (6.21 ms) datasets. Lymph nodes could adequately be detected and measured (Fig 1).

**Fig 2:**
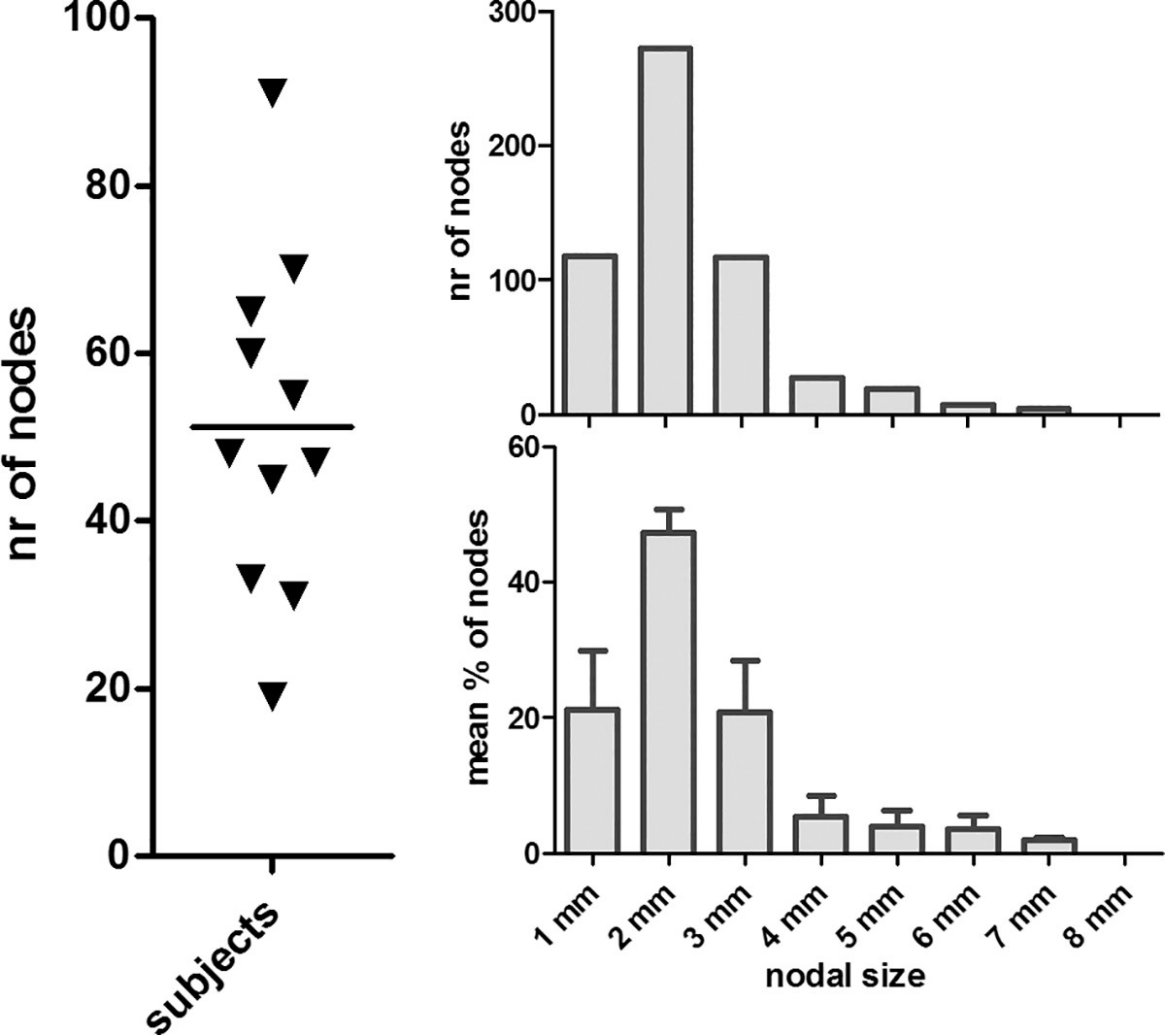
The number and size of pelvic lymph nodes in 11 volunteers. (A) Number of nodes in each subject. (B) Size distribution of 564 lymph nodes. (C) Relative nodal size distribution: the mean (+ SD) of the individual relative size distributions of 11 subjects.

**Fig 3:**
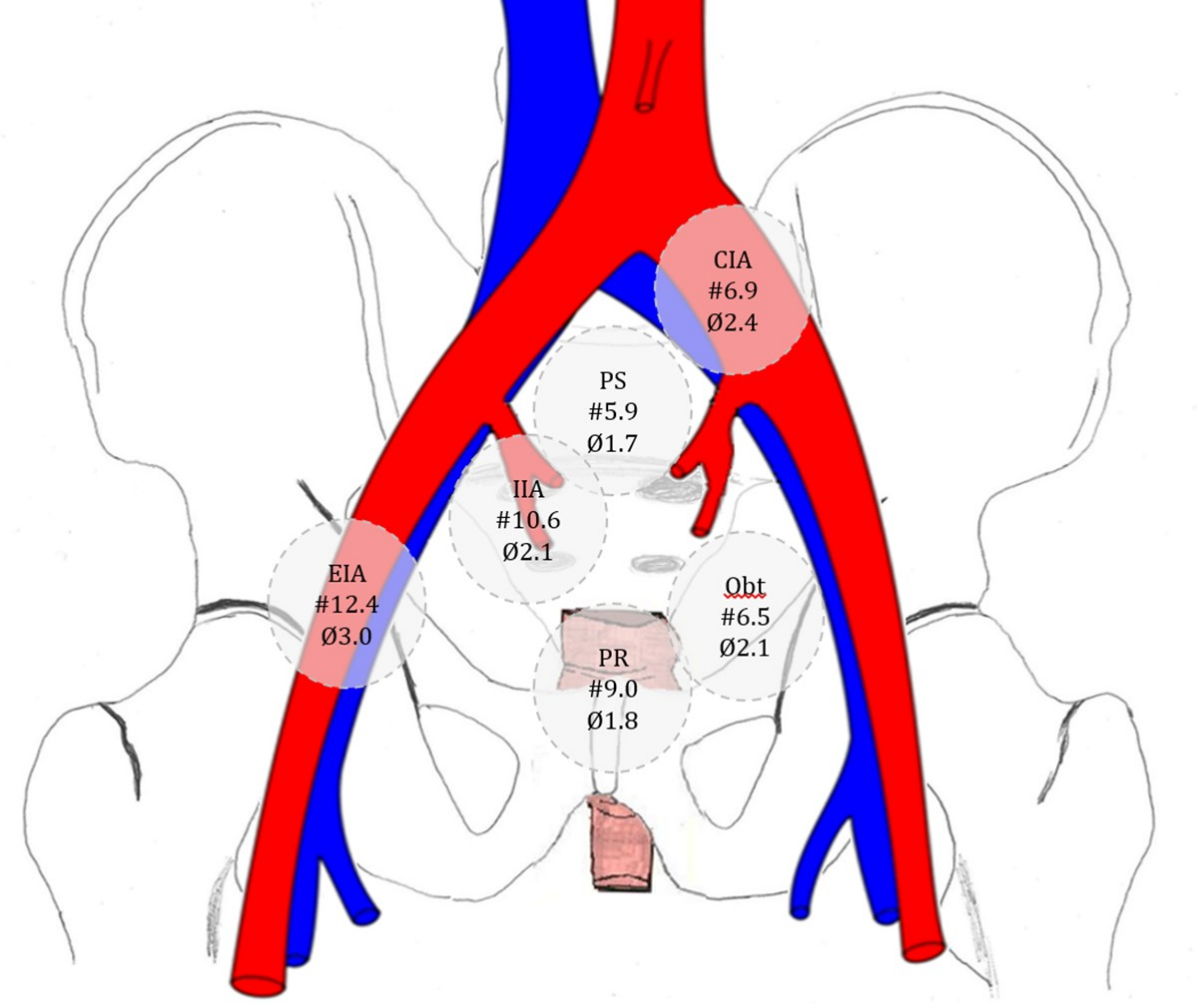
Distribution of pelvic lymph nodes in 11 healthy volunteers. #: Mean number of lymph nodes per region. Ø: mean short-axis lymph node size in millimetres per region. CIA: Common iliac artery region. EIA: External iliac artery region. IIA: Internal iliac artery region. PS: Presacral region. Obt: Obturator region. PR: Pararectal region.

In total 564 lymph nodes were detected in 11 volunteers in the 6 selected regions of the pelvis. The mean number of detected lymph nodes per volunteer was 51 (range 19–91). The measured short axis of the lymph nodes varied from 1 to 7 mm with a mean size of 2.3 mm, varying from a mean size of 1.7 to 3 mm for different locations. Of all detected lymph nodes 69% were 2 mm or smaller. The overall absolute nodal size distribution was very similar to the relative nodal size distribution (normalized to the number of nodes in each subject), apart from small differences in the amount of larger nodes (Fig 2). When normalized, only 14% of the nodes were 4 mm or larger. Most and on average largest lymph nodes were detected in the EIA region (mean number 12.4, mean size 3.0 mm) and least as well as smallest lymph nodes were detected in the presacral region (mean number 5.9, mean size 1.7 mm) (Fig 3, Table 1). Overall median size of the nodes was 2 mm.

**Table 1:** Total and mean amount of lymph nodes in different areas of the pelvis.

Region*	nr nodes	mean nr nodes	range (25% - 75%)	mean size (mm)	range (mm)
CIA	76	6.9	5.5–8.5	2.4	1–5
EIA	136	12.4	7–16	3.0	1–7
IIA	117	10.6	6.5–13.5	2.1	1–4
Presacral	65	5.9	4–7.5	1.7	1–4
Obturator	71	6.5	5–7.5	2.1	1–5
Pararectal	99	9.0	4–13.5	1.8	1–4

*CIA, EIA and IIA are common, external and internal iliac artery regions, respectively.

In two volunteers we experienced some motion artefacts in the upper part of the pelvis. Under-reading of lymph nodes in the common iliac artery region in these patients is therefore possible.

## Discussion

In this work we present an in vivo ultrahigh-field MRI study of the distribution of normal pelvic lymph nodes in volunteers. Typical challenges in homogeneity of body MR images at 7T were overcome and the detection of lymph nodes throughout the pelvis was very well possible.

We detected large numbers of lymph nodes in different areas of the pelvis, with sizes as small as 1 mm. The individually normalized size distributions of nodes did not depend on the number of lymph nodes detected (volunteers with either few or many nodes had similar nodal size distributions), which illustrates a robust detection of lymph nodes down to 1 mm short axis. In compliance with earlier studies [10,21,28–32] we found a large range in the number of detected lymph nodes in the selected regions. Therefore, in our opinion a minimum lymph node count as a stand-alone quality measurement for pelvic lymph node dissections should be reconsidered. This is in concordance with findings in two studies on lymph node involvement in bladder cancer. Meticulous dissection techniques and standardized templates for pelvic lymph node dissection proved to be more important than achieving a minimum lymph node count [33,34].

In this study more lymph nodes were detected on ultrahigh-field MRI in the same regions compared to earlier results on 1.5T MRI [10]. Moreover, we found a smaller mean diameter of lymph nodes. Although no direct comparison between field strengths in the same volunteers was performed, there is a clear indication that more and smaller lymph nodes can be identified with MRI at a higher magnetic field strength. A shared limitation of both our and the Ramirez study is the lack of histology or a confirmation of detected lymph nodes otherwise.

On the other hand, finding more and smaller lymph nodes on ultrahigh field strength MRI is becoming a challenge for radiology-pathology correlation, as is shown in a recent ex-vivo 7T study of the meso-rectum. With standard pathology methods less lymph nodes or lymph node like structures were detected with pathology compared to ultra-high field MRI, even MRI guided. No other water containing structures were revealed as explanation for the water containing spherical structures within the lipid tissue as assessed by MRI [23].

This is for pathology a (not always recognized) challenge. In a review on prostate cancer patients Conti et al [35] describe a shift from traditional handling of lymph node dissection specimens, based on nodal size, identified by palpating the specimens and discarding the rest of the tissue, towards examining all of the removed tissue. To no surprise, in the latter method more lymph nodes were identified [28,35]. Adding serial sectioning of the lymph nodes, immunohistochemistry or the combination of both increased the detection rate of (small) lymph node metastases [22,35].

Furthermore correlation is depending on the dissection specimen provided to the pathologist. Pelvic lymph node dissections can vary from a limited lymph node dissection field to an extended dissection field. In prostate cancer several aberrant locations for metastatic lymph node spread were determined in addition to the standard (extended) dissection field [36,37]. The gold standard of dissection combined with pathology might therefore be not so reliable as always assumed.

Knowledge on number, size and location of lymph nodes contributes to the debate of the therapeutic effect of lymph node dissections or radiotherapy in pelvic cancers [2,3,20,38,39]. Earlier work showed substantial lymph node metastases presence outside the standard (extended) dissection field or whole pelvic radiotherapy (WPRT) field in patients with prostate cancer [36–38,40]. As we show in this paper, ultra-high magnetic field MRI detects very small lymph nodes. It is unlikely that all lymph nodes of these dimensions can be tracked and surgically removed: once metastasized to lymph nodes, surgery with curative intent seems close to impossible. In the event of radiotherapy it is essential to cover all metastatic lymph nodes to create the maximum therapeutic effect. Optimal knowledge on their potential presence and location is therefore crucial.

Earlier results with USPIO-enhanced 1.5T MRI in prostate cancer showed a mean diameter of metastatic lymph nodes of 4.9 mm, with smallest measured lymph node metastasis of 2 mm [20]. The current study shows a mean size of normal lymph nodes on ultrahigh-field MRI of 2.3 mm, 21% of detected lymph nodes being 1 mm. Future challenge is a combination of ultrahigh-field MRI with USPIO or PET for the detection of metastases in these small nodes.

## Conclusions

With advanced MRI using an ultra-high magnetic field it becomes possible to reliably detect small lymph nodes down to 1 mm in the lower abdomen. The range in the number of lymph nodes in different subjects is large, and the mean size and number of nodes differs between regions. With the presented initial results as a nodal anatomical baseline, one could question current validity of representative lymph node dissections and accompanying pathology reports.

When ultra-high magnetic field MRI is combined with a functional imaging technique, a shift of the in vivo detection limits of pelvic lymph node metastases is within reach.
